# Polytrauma in the elderly: predictors of the cause and time of death

**DOI:** 10.1186/1757-7241-18-26

**Published:** 2010-05-13

**Authors:** Nicholas D Clement, Carole Tennant, Cyrus Muwanga

**Affiliations:** 1 Dept. of Trauma and Orthopaedic Surgery, Royal Infirmary of Edinburgh, Little France, Edinburgh EH16 4SU, UK; 2 Dept. of Accident and Emergency, City Hospitals Sunderland NHS Trust, Sunderland Royal Hospital, Kayll Road, Sunderland, SR4 7TP, UK

## Abstract

**Background:**

Increasing age and significant pre-existing medical conditions (PMCs) are independent risk factors associated with increased mortality after trauma. Our aim was to review all trauma deaths, identifying the cause and the relation to time from injury, ISS, age and PMCs.

**Methods:**

A retrospective analysis of trauma deaths over a 6-year period at the study centre was conducted. Information was obtained from the Trauma Audit and Research Network (TARN) dataset, hospital records, death certificates and post-mortem reports. The time and cause of death, ISS, PMCs were analysed for two age groups (<65 years and ≥ 65 years).

**Results:**

Patients ≥ 65 years old were at an increased risk of death (OR 6.4, 95% CI 5.2-7.8, p < 0.001). Thirty-two patients with an ISS of >15 and died within the first 24 hours of admission, irrespective of age, from causes directly related to their injuries. Twelve patients with an ISS of <16, died after 13 days of medical conditions not directly related to their injuries (p = 0.01). Thirty four patients had significant PMCs, of which 11 were <65 years (34.4% of that age group) and 23 were ≥ 65 years (95.8% of that age group) (p = 0.02). The risk of dying late after sustaining minor trauma (ISS <16) is increased if a PMC exists (OR 5.5, p = 0.004).

**Conclusion:**

Elderly patients with minor injuries and PMCs have an increased risk of death relative to their younger counterparts and are more likely to die of medical complications late in their hospital admission.

## Introduction

The elderly population within the United Kingdom continues to grow [1] and hence we are encountering more elderly patients who have suffered trauma. Despite this growing number of elderly patients they form a small percentage of trauma patients overall, but they do consume a disproportionate amount of medical resources [2] and are more likely to require admission to hospital [3]. However, aggressive early management of such patients results in an increased survival rate, and of those who survive the majority return home [4]. Mortality due to trauma continues to fall, but the highest rates are seen in those patients older than 65 years of age, for which the majority are secondary to falls [5].

Elderly patients and in particular those with pre-existing medical conditions (PMC) have been demonstrated to be at an increased risk of mortality after incurring injuries of minor to moderate severity [6]. A suggested explanation for this is the physiological changes associated with aging e.g. diminished respiratory and cardiovascular reserve/function, PMC and medications [7]. This diminished physiological reserve is thought to result in elderly patients being less able to respond to a traumatic insult and hence their worse prognosis. Not only are elderly patients at an increased risk of mortality, but they die later in their admission [8]. It is also known that the rate of medical complications is higher in non-survivors [8].

To date, the association between the cause and time of death after trauma and the relation to age, injury severity and the association of PMC are not known. We reviewed all trauma deaths for a defined period, identifying the exact cause of death and its relation to injury severity, the affect of age and PMCs.

## Patients and Methods

A retrospective review of trauma deaths over a 6 year period (2000 to 2006) at the study institute was conducted. The study institute is a district general hospital with a patient population of 300,000 [9] and is the only hospital for the catchment area receiving all trauma patients. Our unit has prospectively submitted trauma data to the Trauma Audit and Research Network (TARN) group since 2000, employing a trained data analyst to compile and submit all information, of which completion is of high quality [10]. Trauma deaths were identified using the TARN dataset and their hospital records were reviewed. Information was obtained from the TARN dataset, with regard to national, other district general hospitals and neurosurgical centers, allowing a comparison to be made with the study institute.

TARN was founded in 1989 and is an on going trauma epidemiology study [11]. Approximately half of all trauma-receiving hospitals in England and Wales currently submit information regarding the trauma patients they manage. Criteria for submission to this data set are: 1. Length of hospital stay of 72 hours or more, 2. Transfer to specialist centre for extended trauma care, 3. Admission to intensive care, or 4. Death occurring in hospital irrespective of cause. Patients with an isolated simple injury, including fracture neck of femur in those greater than 65 years, are excluded from the database. Each patient has an injury severity score (ISS), assigned according to their anatomic injury and a predicted survival score assigned by trained coders at the network centre [10]. TARN has ethical approval for research on anonymised data through the patient information access group (PIAG3-04(E)2006).

**Figure 1:**
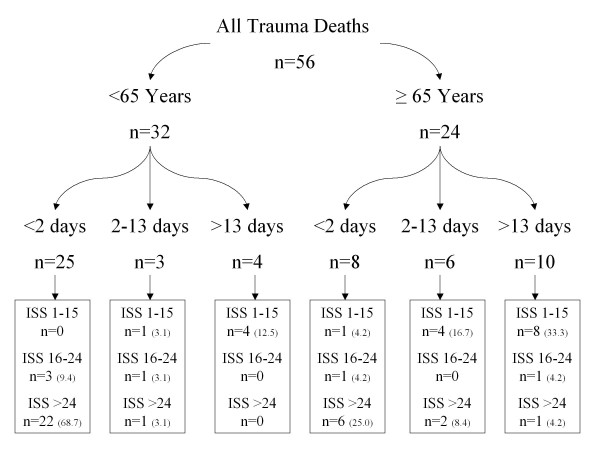
**Flow diagram for the 56 deaths divided according to age, time from injury and ISS (% for age group)**.

Previous authors have defined their elderly population as those patients aged at least 65 years of age [4,8]. Giannoudis et al demonstrated a variation in time of death after injury between the younger and older age groups [8]. The deaths were segregated into two groups <65 years and ≥ 65 years and was further divided into three subgroups relating to time of death after admission (Figure 1): <2 days, 2-13 days and >13 days. ISS were stratified into three range groups (ISS: 1-15, 16-24, >24) for each age group and analysed according to the time of death. The cause of death that was given on the death certificate was recorded, and confirmed or refuted with a postmortem report, if carried out.

PMCs were identified from the TARN dataset and confirmed and amended as appropriate on review of the medical notes. PMC(s) are recorded as free text and coded by the TARN office using a list of common conditions, which has previously been described [6]. Those patients with no PMCs were identified as "none".

SPSS 16 software was used for statistical analysis [12]. Mann-Whitney U tests were used to compare those with and without PMCs for age and ISS. Dichotomous variables were assessed using Fishers exact test. Multiple logistic regression analysis was used to predict mortality adjusting for ISS, age and hospital type. Statistical significance was assumed at the p < 0.05 level. Primary outcome was time and cause of death according to age, ISS and PMC.

## Results

### Comparability of data

Analysis of the demographic data and ISS for both national and the study centre revealed no statistical significant difference. However, a difference was observed in the ≥ 65 years group (Table 1), the study centre cohort had a greater proportion of patients suffering an ISS of 16 to 24 and a female preponderance. A significant difference was observed for the ISS and predicted survival scores between the <65 year olds and those ≥ 65 years old, with a mean score of 30.7 versus 20.5 and 41% versus 47% respectively (p < 0.001).

**Table 1:** Case mix comparison of patients ≥ 65 years for both the TARN dataset and the study centre (SC).

	SC	TARN	p-value
**Median Age**	76.4 Yrs	77.6 Yrs	0.9

**25^th ^Percentile**	71.4 Yrs	70.6 Yrs	0.8

**75^th ^Percentile**	83.7 Yrs	82.3 Yrs	0.7

**Females**	76.7%	70.5%	0.05

**ISS 1-8**	18.6%	20.1%	0.9

**ISS 9-15**	67.5%	74.3%	0.5

**ISS 16-24**	9.3%	3.0%	0.05

**ISS 25-40**	3.6%	1.9%	0.2

**ISS >40**	0.9%	0.6%	0.3

Using all other district general hospitals submitting data to TARN as a baseline the odds of death within the study centre adjusting for age and ISS were significantly reduced (OR 0.7, 95% CI 0.52-0.97 p = 0.03).

### Study cohort

One thousand nine hundred and twenty patients were submitted from the study centre to the TARN dataset during the study period. There was no significant difference observed for admission and inter-hospital transfer between the study centre and other district general hospitals, but the readmission figures are significantly lower for our centre (p = 0.03).

### Mortality

There were 56 deaths, of which 24/462 (5.2%) were ≥ 65 years and 32/1458 (2.2%) were <65 years (p = 0.01). The average annual mortality rate for all units submitting data to TARN (excluding the study centre) was 5.8% (5316/92084) and for the study centre was 2.9%. Three patients, all of which were aged <65 years old, sustained penetrating injuries and all of died within 24 hours of admission. Table 2 illustrates the unadjusted mortality according to age group for both national units and the study centre.

**Table 2:** Unadjusted mortality according to age for TARN and study centre (SC).

	TARN ≥ 65	SC ≥ 65	TARN <65	SC <65
**Survivors**	18845	462	59333	1458

**Dead**	2408	24	12362	33

**Mortality Rate %**	11.3	5.2	7.2	2.3

### Age, time of death and ISS

**Figure 2:**
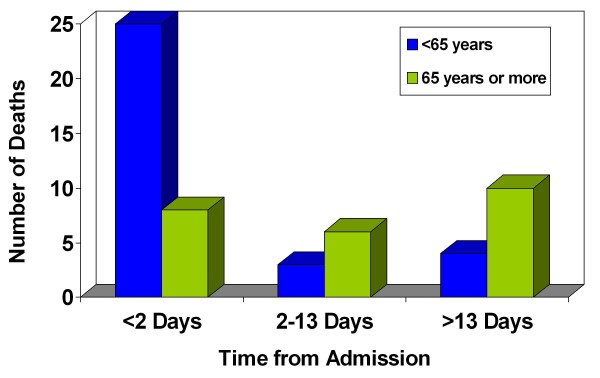
**Pattern of mortality in relation to age and time from admission**.

Thirty-two patients (57.1% of all patients) with an assigned ISS of >15 died within the first 24 hours of admission to our unit (Figure 1). The cause of death was directly related to the trauma insult. Twelve patients (21.4% of all patients) with an assigned ISS of <16, died after 13 days from admission, of which all died of medical conditions not directly related to their injuries (Table 3). The pattern of death in relation to age and time from admission is summarised in Figure 2, from which two peaks are observed: early (<2 days) and late (>13 days). The early peak mainly consists of those patients <65 years, but this is reversed in the late peak with the majority of patients being ≥ 65 years (p = 0.01).

**Table 3:** Patients who died beyond 13 days with an ISS of <16.

Patient Age Yrs	ISS	Predicted Survival	Stay	Cause of Death as per Death Certificate*
38	4	99%	22 days	1a Bilateral PE 1b Antemortem thrombosis of popliteal vein

53	9	98%	21 days	1a Bronchopneumonia 2 Femur fracture, Korsakoffs disease

56	9	97%	34 days	1a Pneumonia 1b Fractured neck fracture 2 Alcohol excess, Epilepsy

62	9	99%	29 days	1a Acute Myocardial infarction 1b Arteriosclerosis 1c Coronary Heart Disease

69	9	98%	15 days	1a Left basal pneumonia 1b COPD

71	9	98%	24 days	1a COPD 1b Bronchopneumonia 1c Pneumothorax 2 Fractured neck of humerus and metatarsal bilaterally

77	9	92%	24 days	1a Bronchopneumonia

78	9	94%	40 days	1a Non Hodgkin's lymphoma

79	9	95%	26 days	1a Left ventricular failure 1b Ischaemic heart disease 2 Sigmoid cancer 2b COPD

84	8	93%	55 days	1a Respiratory failure 1b Pneumonia

87	9	92%	15 days	1a Congestive cardiac failure 1b Bronchopneumonia 1c COPD

90	9	94%	33 days	1a Left ventricular failure 2 Fractured left shaft of femur

*A death certificate in the UK is completed by a medical doctor and has two parts: part 1 being the cause of death which is split into a (1a), being the exact cause, b (1b) the disease that lead to a, and c (1c) the disease that lead to b, part 2 is contributing, but not directly causing death.

COPD = Chronic Obstructive Pulmonary Disease

**Figure 3:**
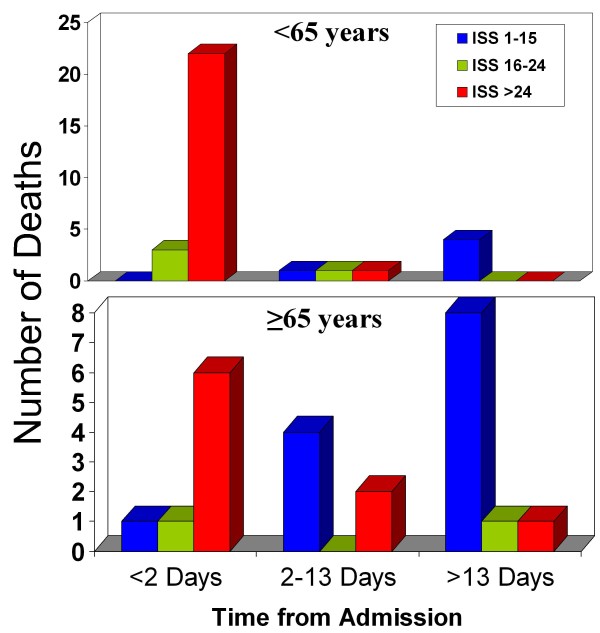
**Deaths of patients <65 and ≥ 65 years of age in relation to time from admission**.

Twenty two patients <65 years (68.7% of that age group) died within 24 hours of admission after suffering an ISS of >24, with only 4 (12%) patients dying beyond 13 days after suffering an ISS <16 (Figure 3). In contrast for patients ≥ 65 years only 6 (25% of that age group) died within 24 hrs of admission after suffering an ISS of >24, but 8 (33.3%) patients died beyond 13 days after suffering an ISS <16 (Figure 3). This difference was statistically significant (p = 0.01). Figure 1 summarises the time of death after admission and ISS stratification for each age group.

Using the entire TARN dataset the risk of death was found to relate to the ISS, with a score of 1 to 8 taken as a baseline, using multivariate analysis (age, gender, PMC). The odds of death increased with worsening ISS:

• 9 to 15 OR 1.2, 95% CI 0.85-1.6, p = 0.333

• 16 to 24 OR 7.4, 95% CI 5.2-10.6, p < 0.001

• >24 OR 52.4, 95% CI 37.4-7.8, p < 0.001

Mortality was associated with age, those patients ≥ 65 years old were at an increased risk of death relative to those <65 years old (OR 6.4, 95% CI 5.2-7.8, p < 0.001).

### PMC

Thirty four patients had significant PMCs, of which 11 were <65 years (34.4% of that age group) and 23 were ≥ 65 years (95.8% of that age group) (p = 0.02). There were 12 patients in total that died beyond 13 days who had sustained relatively minor injuries (ISS<16), all of which had PMC. The majority (66.6%) of this group consisted of patients' ≥ 65 years, and three of those <65 years were >50 yrs with multiple PMC. Hence, the risk of dying late after sustaining minor trauma is increased if a PMC exists (OR 5.5, p = 0.004). Of the twelve that died beyond 13 days with minor trauma the commonest cause of death was pneumonia (6/12, 50%). Each patient received a physician review during his or her admission. All but one had deterioration of physiological observations 72 hours prior to death, however death was not averted despite medical intervention.

## Discussion

We have shown that patients with minor injuries (ISS <16) and PMC are at an increased risk of late death from medical complications that are not directly related to their original injury.

The transfer of patients out with the study centre may be the reason for the overall low mortality observed in our unit. However, many district general hospitals have the same transfer arrangement and had a higher mortality rate during the study period [10]. We believe our units care is equal to that of any district general hospital and our case-mix is similar to that experienced across the UK and Europe.

Those patients with a high ISS died early during their admission (<48 hours) and those with a low ISS died late in their admission (>13 days). Patients that died early did so because of their trauma insult which is reflected by a high ISS, but those dying late do so because of medical complications. These medical complications in our cohort occurred in patients with significant PMC's, of which the majority were ≥ 65 years old. It is already known that mortality is predicted by ISS and medical complications in older patients [13], of which infections and chest complications are twice as common and dysrhythmias five times more frequent [14]. Age alone has been illustrated as an independent risk factor for mortality [15]. Also, the presence of PMC increases the odds of experiencing a complication to over threefold [16]. The combination of age and PMC is additive, with worsening mortality risk [6].

Hollis et al have demonstrated that PMCs and increasing age are independent risk factors of mortality after trauma [6]. However, this increased risk diminishes with escalating ISS and is no longer statistically significant at with scores >24, which may suggest the trauma insult causes death before medical complications ensue. This trend could be due to these risk factors predisposing them to medical complications, which are not directly related to their trauma insult, after sustaining minor to moderate injuries. Our study supports this theory, demonstrating irrespective of age after sustaining a severe trauma insult the majority who die do so within 48 hours, but elderly patients with PMC die of medical complications not directly related to the initial trauma insult late in their admission after suffering injuries that they may be expect to survive with low ISS and high predicted survival scores. It may be that early medical/physician intervention may avert these late deaths due to medical complications, and patients older than 65 years with PMC could be targeted with early physician input.

Early intensive monitoring, evaluation and resuscitation of elderly patients improve survival after trauma [17]. This costly medical support is justified with few requiring nursing home care on discharge and the majority returning home [18]. It was first suggested by Richmond et al that a care of the elderly consultation service could be an important addition to the trauma team, optimising PMC and managing medical complications that arise [16]. This was confirmed by Fallon et al who demonstrated improved medical care in elderly patients after review by a physician, addressing new and existing medical issues and reducing hospital acquired complications, such as functional decline, falls, delirium and death [19].

A recent study comparing the differences between severely (ISS >15) injured patients older that 65 years old and those less than 65 years old found that in contrast to younger patients despite normal physiological parameters on admission the older age group was at an increased risk of inpatient mortality [8]. Due to this phenomenon the authors suggest it may be difficult to predict which older patients would benefit from aggressive monitoring and management. The authors hypothesise the observed discrepancy may relate to PMC, which were not analysed in their study. Our study supports this theory identifying those patients with PMC being at an increase risk of inpatient mortality that is independent of age, and so would need to be accounted for in statistical analysis of physiological parameters. They also observed that older patients tended to die later in their admission, a trend that we have also demonstrated.

The question remains of how we can identify those patients with an increased risk of death late in their admission after sustaining minor to moderate trauma? Skaga et al described using the American Society of Anesthesiologists (ASA) Physical Status classification to predict mortality, finding it to be an independent predictor [20]. We retrospectively assigned a pre-injury ASA score [21] to the 12 individuals that died beyond 13 days after injury with an ISS of <16. All except one had an ASA score of 3, which is associated with an increased risk of mortality (adjusted OR 2.25) [20]. More specifically for patients with an ISS of <16, mortality increases from <1% in those with an ASA grade of one to approximately 8% in those with as ASA grade of 3 or 4.^20 ^In conjunction with other risk factors for morality, the ASA grade could be used to identify those individuals most at risk and early intervention may avert later death.

## Conclusion

Elderly patients with minor injuries and PMCs have an increased risk of death relative to their younger counterparts and are more likely to die of medical complications late in their hospital admission.

## Conflict of interests

The authors declare that they have no competing interests.

## Authors' contributions

CT complied the patient information for the TARN database, and identified all patients involved in this study. Furthermore she retrieved all death certificates and post-mortem reports. SM was the senior author and gave direction for the study, being the TARN director at the study institute. He was also involved in editing final composition of the paper. NC reviewed all notes and performed statistical analysis, and was the main author of the paper. All authors have read and approved the final manuscript.

## References

[B1] Office of national statistics http://www.statistics.gov.uk/cci/nugget.asp?id=949 date last accessed 17^th ^April 2010

[B2] MacKenzie EJ Morris JA Smith GS Fahey M Acute hospital costs of trauma in the United States: implications for regionalized systems of care Journal of Trauma 1990 30 1096 103 2213943 10.1097/00005373-199009000-00005

[B3] Court-Brown CM Clement N Four score years and ten An analysis of the epidemiology of fractures in the very elderly Injury 2009 40 1111 4 10.1016/j.injury.2009.06.011 19596316

[B4] Broos PL D'Hoore A Vanderschot P Rommens PM Stappaerts KH Multiple trauma in elderly patients. Factors influencing outcome: importance of aggressive care Injury 1993 24 365 8 10.1016/0020-1383(93)90096-O 8406738

[B5] Griffiths C Wright O Rooney C Trends in injury and poisoning mortality using the ICE on injures statistics matrix, England and Wales, 1979-2004 Office for National Statistics 2006 1 14 17165466

[B6] Hollis S Lecky F Yates DW Woodford M The effect of pre-existing medical conditions and age on mortality after injury Journal of Trauma 2006 61 5 1255 60 10.1097/01.ta.0000243889.07090.da 17099538

[B7] Morris JA MacKenzie EJ Damiano AM Bass SM Mortality in trauma patients: the interaction between host factors and severity Journal of Tauma 1990 30 1476 82 2258958

[B8] Giannoudis PV Harwood PJ Court-Brown CM Pape HC Severe and multple trauma in older patients; incidence and mortality Injury 2009 40 362 7 10.1016/j.injury.2008.10.016 19217104

[B9] 2001 Census Office for National Statistics http://www.statistics.gov.uk/census2001/pyramids/pages/00cm.asp date last accessed 17^th ^April 2010

[B10] Trauma Audit and Research Network http://www.tarn.ac.uk/Content.aspx?c=2906 date last accessed 17^th ^April 2010

[B11] Trauma Audit and Research Network http://www.tarn.ac.uk date last accessed 17^th ^April 2010

[B12] SPSS SPSS for Windows, version 16.0.1 2009 Chicago: Lead Technologies

[B13] Tornetta P Mostafavi H Riina J Turen C Reimer B Levine R Behrens F Geller J Ritter C Homel P Morbidity and mortality in elderly trauma patients Journal of Trauma 1999 46 4 702 6 10.1097/00005373-199904000-00024 10217237

[B14] Schiller WR Knox R Chleborad W A five-year experience with severe injuries in elderly patients Accident, Analysis and Prevention 1995 27 2 167 74 10.1016/0001-4575(94)00053-O 7786384

[B15] Taylor MD Tracy JK Meyer W Pasquale M Napolitano LM Trauma in the elderly: intensive care unit resource use and outcome Journal of Trauma 2002 53 3 407 14 10.1097/00005373-200209000-00001 12352472

[B16] Richmond TS Kauder D Strumpf N Meredith T Characteristics and outcomes of serious traumatic injury in older adults Journal of the American Geriatric Society 2002 50 2 215 22 10.1046/j.1532-5415.2002.50051.x 12028201

[B17] Demetriades D Karaiskakis M Velmahos G Alo K Newton E Murray J Asensio J Belzberg H Berne T Shoemaker W Effect on outcome of early intensive management of geriatric trauma patients British Journal of Surgery 2002 89 10 1319 22 10.1046/j.1365-2168.2002.02210.x 12296905

[B18] DeMaria EJ Kenney PR Merriam MA Casanova LA Gann DS Aggressive trauma care benefits the elderly Journal of Trauma 1987 27 11 1200 6 3682032 10.1097/00005373-198711000-00002

[B19] Fallon WF Jr Rader E Zyzanski S Mancuso C Martin B Breedlove L DeGolia P Allen K Campbell J Geriatric outcomes are improved by a geriatric trauma consultation service Journal of Trauma 2006 61 5 1040 6 10.1097/01.ta.0000238652.48008.59 17099506

[B20] Skaga NO Eken T Sovik S Jones JM Steen PA Pre-injury physical status classification is an independent predictor of mortality after trauma Journal of Trauma 2007 63 5 972 8 10.1097/TA.0b013e31804a571c 17993938

[B21] ASA Physical Status Classification System American Society of Anesthesiologists http://www.asahq.org/clinical/physicalstatus.htm date last accessed 17^th ^April 2010

